# Principle of relationship between seepage movement and action

**DOI:** 10.1038/s41598-022-21335-9

**Published:** 2022-11-03

**Authors:** Zhiwang Diao, Changyi Wang, Keqiang He, Lu Guo

**Affiliations:** 1 School of Civil Engineering, Qingdao University of Technology, No. 777 Jialingjiang East Road, Huangdao District, Qingdao, 266520 Shandong China; 2 Shandong Yantai Penglai Natural Resources and Planning Bureau, No. 191, Beiguan Road, Penglai District, Yantai, 264000 Shandong China; 3 Department of Civil Engineering and Architecture, Suqian University, Jiangsu, China

**Keywords:** Environmental sciences, Hydrology

## Abstract

The seepage is completed under the control of the action. There is a definite relationship between the seepage and its controlling factors. The recharge flow, seepage flow and water level change are unified. In this paper, the exact and complete relationship among seepage flow, recharge flow and water level change is discussed in detail through field observation and repeated tests, and a new equation for clarifying seepage flow law and water level change law is established, it provides more accurate, more convenient and more reliable theoretical basis for solving the problem of seepage flow. At the same time, through the study of seepage movement law, it is found that the method of parameter introduction has some disadvantages, and then a new method is established to study the law of seepage movement.

## Introduction

In January 2020, Hans Publishing House published the book *Principles* *and* *Laws* *of* *Action*^1^. In this book, the relationship between fluid movement and its control action is expounded, and it is pointed out that there is a unified relationship among seepage, stagnation and action, which opens up a new direction for the development of seepage mechanics^2^. However, the description of the relationship among seepage, stagnation and action in the book is not accurate enough. In spite of this, action science has played a great role in promoting the progress in the field of seepage mechanics.

Seepage mechanics is an applied science focusing on the quantitative study of the unified relationship between seepage and its control factors. Seepage mechanics came into being in 1856 when French engineer Darcy published the linear law of seepage in uniform sand layer, namely Darcy's law^3^. Darcy's research is the only one in the history of seepage mechanics that focuses on the relationship between seepage and its control factors, which has important scientific and historical significance. However, Darcy's law is not perfect. The specific defects are as follows: (1) The unified problems of recharge flow, seepage and water level rise and fall are not considered, which leads to the inaccurate and incomplete description of the seepage law by Darcy's law, which forms an obstacle to the reasonable solution of practical problems. (2) Darcy's law is experimentally deduced by using the parameter introduction method. On the basis of considering that seepage is proportional to head pressure and inversely proportional to seepage distance, Darcy's law is established by introducing adjustment coefficient, and the relationship among seepage, head pressure and property parameters of seepage channel is not discussed. (3) The property parameters of seepage channel are actually equivalent to the percentage of leakage and belong to dimensionless parameters, but the permeability coefficient in Darcy's law is a dimensional parameter. (4) Darcy's law is lack of applicability in practical application.

Darcy's law emphasizes that the head pressure is proportional to the seepage rate, but ignores other related factors.Therefore, the seepage is not only controlled by the water level pressure, but also affected by many other factors.

Darcy's law states that the power source to control the movement of groundwater is only the head pressure. However, the water level pressure is caused by stagnant water, which is controlled by the injection of recharge flow and the hindrance of obstacles. Therefore, the first dominant factor in the seepage phenomenon is the existence of recharge flow. Without recharge flow, there will be no stagnant water level, and there will be no seepage flow. Although stagnant water can in turn produce the driving force of seepage, it is a secondary role, not a dominant role.

More than 160 years after Darcy's law was established, the development direction of seepage mechanics is mainly mathematical analysis methods. In 1863, Dupuit put forward the famous one-dimensional steady seepage flow hypothesis, he believed that the vertical component of groundwater velocity can be ignored when phreatic water flows slowly, and further extended Darcy's law to solve practical problems. Qian (1958) proposed an electrical simulation method for solving the seepage field, which is used to analyze more complex seepage problems. With the rapid development of computer technology, numerical analysis method is widely used in seepage analysis. Finite difference method, finite element method and boundary element method have gradually become common seepage numerical analysis methods. Peng et al. proposed the modified water capacity and accelerated iterative convergence method^4^. Li et al. introduced the permeability coefficient as a random field into the functional variational expression, deduced and established the stochastic variational principle and corresponding stochastic finite element formulation of three-dimensional stable seepage by using the small parameter perturbation method, and derived the mean and variance expressions of water head and hydraulic gradient^5^. Okeke et al. found that the stability of slope was significantly influenced by permeability coefficient, pore shape, hydraulic conductivity and the amount of gravel and pebbles in the slope^6^. Tian et al. used a new unsaturated soil seepage measurement equipment to study the stable seepage of unsaturated loess^7^. The results showed that the lower the water content, the smaller the permeability coefficient of unsaturated soil, and the longer the time required for the seepage to reach the steady state. Huang et al. considered the confining pressure and interface roughness, and studied the evolution laws of hydraulic gradient and hydraulic shear stress under seepage through a series of experiments^8^.The effect of seepage on the stability of geotechnical engineering, such as natural slopes, dams, and tunnel construction, is constantly being mentioned. Lee and Nam studied the seepage problem caused by groundwater flowing into the tunnel^9^. Ghiassian and Ghareh proved by experiments that the stability of shallow block sliding in saturated sandy slopes under seepage depends on the flow direction and hydraulic gradient^10^. Koltuk and Azzam showed through numerical analysis and model tests that quicksand conditions that do not require three-dimensional failure bodies can be used to evaluate the foundation stability of slab excavation pits against seepage failure in cohesive soils^11^. Koltuk et al. conducted experimental and numerical studies to elucidate seepage failure in tabular excavation pits in layered cohesive soils^12^. In the last 20 years, all branches of seepage mechanics have developed. Unfortunately, the basic relationship between seepage and its control factors has been stagnant.

In this paper, the understanding of the law of fluid motion is not produced in the special study of fluid motion, but in the process of applied research of action science. In order to demonstrate the correctness of action theory, researchers have carried out observation and experimental analysis in many fields to prove the correctness and practicability of action theory. The research on the law of fluid motion in this paper is one of them.

Around 1986, the basic theory of functionology had been basically determined, but because of the conflict between its basic theory and the existing science, many people do not accept it, and the functional theory is too partial to the theory, so it is difficult to publish its research results in time. In this context, functionology began to be used in some fields of applied research and demonstration. Because the applied research of action science involves the demonstration of some laws and questions about its defects, this leads to all kinds of pressure and seriously hinders the progress of action science. During the period from 1986 to 2010, the applied research of action science was mainly involved in the fields of fluid movement, landslide prediction, deformation and so on. The core of pragmatics is mainly composed of relation equations between action and force, action equations, change equations, action and change unified equations.

In 2021, the unified research on seepage, stagnation and action has been further improved. This paper mainly describes the final results of this research.

### Basic law of seepage flow

The fluid itself does not have the ability to permeate or flow, and it is because of the acceptance of the fluid that it has the ability to flow and percolate. The basic law of seepage movement is that the fluid receives one or more driving effects, and then percolate in the material and space environment that can percolate, or accumulates in the space and material conditions that cannot be excreted. Driving action is the main factor of seepage phenomenon, and fissures, pore space and substances that prevent seepage constitute the restricting factors to control the operation and non-operation of seepage. Therefore, in the process of studying and understanding the seepage phenomenon, the focus is on the relationship between the nature of the environment and the seepage. The problem is how to correctly understand and master the basic law of seepage.

In order to find the answer, we first seek the basic law of seepage flow from the objective world. When the atmosphere rain water falls to the ground, part of the water seeps into the ground in the permeable area, and the other part forms the surface slope flow under the control of the topographic slope, and then converges into the river. Part of the surface flow and rivers infiltrate into the ground when they encounter permeable areas during the flow process, and the other part continues to flow. When the surface flow is blocked by the raised surface, stagnant flow is formed in the blocked area, resulting in stagnant water and rising water level. If there are permeable pores underground in the stagnant area, the water collected in the stagnant water area will still seep underground. When the pooling recharge flow is greater than the infiltration discharge, the two different physical quantities of infiltration discharge and water level rise will be observed or detected at the same time; when the recharge flow is less than the infiltration discharge, two different physical quantities of infiltration discharge and water level drop can be observed or detected; when the recharge flow disappears, the stagnant water still produces seepage flow and water level will decline until the stagnant water disappears.

According to this observation phenomenon, we can draw a conclusion that three phenomena of recharge flow, seepage flow and stagnant flow coexist in the process of water flow movement. So, it is necessary to understand the leading role of the simultaneous occurrence and unity of the three phenomena of water supply movement, seepage movement and stagnant water movement. Understanding and mastering the law of action is the key. It is concluded that the study of seepage law can be carried out from two aspects: the whole seepage law and the local seepage law.

### Study on the whole law of the whole seepage system

Paying attention to the whole seepage system is to take the whole seepage system as the research object and understand the basic law of seepage as a whole. The advantage of this research perspective is that it can eliminate the one-sidedness of research understanding. The complexity of the seepage system needs to be studied comprehensively to understand its overall law.

To study the role of controlling seepage, we first determine a basic acting surface. From the overall point of view, the seepage entrance section or seepage passage is generally regarded as the affected section, and the discharge outlet section can also be taken as the object of study. If the discharge outlet is the contact surface between the mechanical well and the aquifer, the contact surface can also be used as the object of study. The driving force acting on the cross section comes from three aspects: the initial momentum of the recharge flow itself, the momentum increment caused by gravity, and the action of the initial stagnant water.

If the recharge flow is *Q*, the momentum of *Q* will be transferred to the seepage affected section, and then the action value of the initial momentum of the replenishment flow *A*_1_ can be determined by Eq. (1).

1 $$A_{1} = \rho \frac{{Q^{2} t}}{S}$$

where: $\rho$ is the material density of the recharge flow; *S* is the area of the affected section; t is the operation time of recharge and seepage. The initial velocity of recharge flow is *v* = *Q/S.*

When *Q* enters the seepage action system, it continues to be affected by gravity, Thus, the action amount on the seepage section *A*_2_ can be obtained, and its value can be determined according to Eq. (2).

2 $$A_{2} = \int_{0}^{t} {\rho Qgt} dt$$

where: *g* is the acceleration of gravity; *m* = $\rho Qt$ is the quality of recharge flow; *v* = *gt* is the velocity increment of the recharge flow.

If there is ponding above the section, the pressure of this part of ponding will also act on the affected section, this action is the head pressure action in Darcy's law, and its magnitude of the action can be determined according to Eq. (3).

3 $$A_{3} = \rho V_{0} gt$$

The action quantity controlling seepage and stagnant flow operation is synthesized by these three actions, as shown in Eq. (4). Whether seepage or stagnant flow depends on the nature of seepage and stagnant flow operation environment. If there is no seepage channel, there is no seepage flow, only ponding water increment and water level increment; if the seepage channel is unblocked, the seepage flow is large and the stagnation flow is small; on the contrary, it is also the opposite.

4 $$A = \rho Q\left( {\frac{Q}{S} + gt} \right)t + \rho V_{0} gt$$

where: *V*_0_ represents the volume of the initial stagnant ponding water. *A* is the action quantity of controlling seepage operation.

The action *A* is divided into two parts by the seepage section: the virtual action A_F_ and the real action *A*_*T*_, which are *A*_*F*_ = *EA* and *A*_*T*_ = *TA* respectively. Among them, *A*_*F*_ leads to changes in seepage, and *A*_*T*_ causes changes in stagnant water. The quantity of seepage change is called the seepage increment, and its value is *B*_*F*_=$\rho$ *q*^2^*t/ES*, *ρ* is the seepage flow, *E* is the porosity of the seepage section. The quantity of the ponding water change is called the ponding increment, and considering the unity of dimensions, its value is *B*_*T*_ = *ρ(△V)gt*, and *△V* is the water volume increment. The study knows that:

5 $$A_{F} = E\left[ {\rho Q\left( {\frac{Q}{S} + gt} \right)t + \rho V_{0} gt} \right] = B_{F} = \frac{{\rho q^{2} t}}{ES}$$

6 $$A_{T} = T\left[ {\rho Q\left( {\frac{Q}{S} + gt} \right)t + \rho V_{0} gt} \right] = B_{T} = - \rho \left( {\Delta V} \right)gt$$

### Study on the local law of seepage

Focusing on the study of the seepage movement of any local section, only the water level difference between the upper and lower water level of the seepage section and the relationship between recharge and seepage through the section are considered, and the factors such as remote recharge and gravity are not considered. This kind of research which focuses on the local relationship between the upper and lower sections has the defects of limited vision and easy to make one-sided mistakes, but it is convenient for the application of mathematical analysis methods.

**Figure 1:**
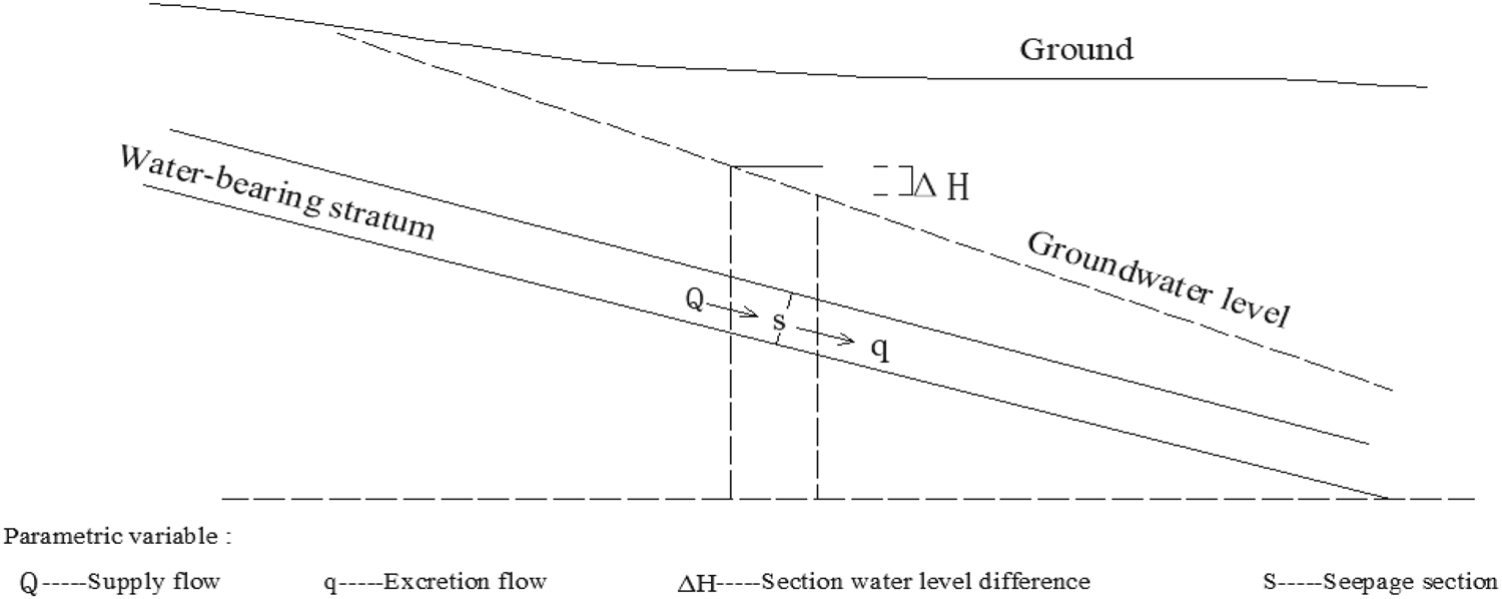
Schematic diagram of the relationship among water level difference, recharge flow and seepage flow.

As shown in Fig. 1, the water in an underground aquifer seeps from high to low under pressure, any seepage section area is *S*, the flow into section *S* is *Q*, the flow rate from section *S* is *Q*, and the water level difference between the top and bottom of the section is △*H*. The seepage parameter of the section is *E*, the impermeable parameter is *T*, and the length of the section is *b*. So, the following functional relationship exists between these two quantities.

The action *A*, which is produced by the action of flow *Q* on section *S*, *A* = *ρQ*^*2*^*t/S*. Where the action used to overcome resistance is *A*_*T*_ = *T* $\rho$ *Q*^*2*^*t/S,* called real action. *T* is called blocking rate, the momentum running through the cross section is called virtual action, and its value is *A*_*E*_ = *E* $\rho$ *Q*^*2*^*t/S.* It is called virtual action. *E* is the pass rate. The blocking property of the seepage channel has an blocking effect. The amount of resistance is equal to the resistance *R* multiplied by the time *t*, that is *A*_*R*_ = *Rt*. The relationship between the amount of implementation and the amount of hindrance is *A*_*T*_ = *−* *A*_*R*_. The blocking action produces stagnant water momentum, that is, stagnant water or stagnant water. The phenomenon of stagnant water is regarded as a real change phenomenon. The value of the real change is *B*_*T*_ = *−*$\rho$*(△H)bgt,* *B*_*T*_ represents the real change, *△H* is the water level increment. *b* is the width of the cross section of the flow channel. The momentum lost through the cross-section seepage is *B*_*F*_ =$\rho$ *q*^*2*^*t/ES,* *B*_*F*_ is called virtual change. The study knows that:

7 $$A_{F} = E\frac{{\rho Q^{2} t}}{S} = B_{F} = \frac{{\rho q^{2} t}}{ES}$$

8 $$A_{T} = T\frac{{\rho Q^{2} t}}{S} = B_{T} = \rho \left( {\Delta H} \right)bgt$$

It can be seen that the overall law of seepage is consistent with the local law.

## Summary and application of the unified law of seepage and action

The unified relationship between seepage and its control is of great value in practice. Then, how to apply the unified law is discussed in detail here, and its application methods are as follows.

**Figure 2:**
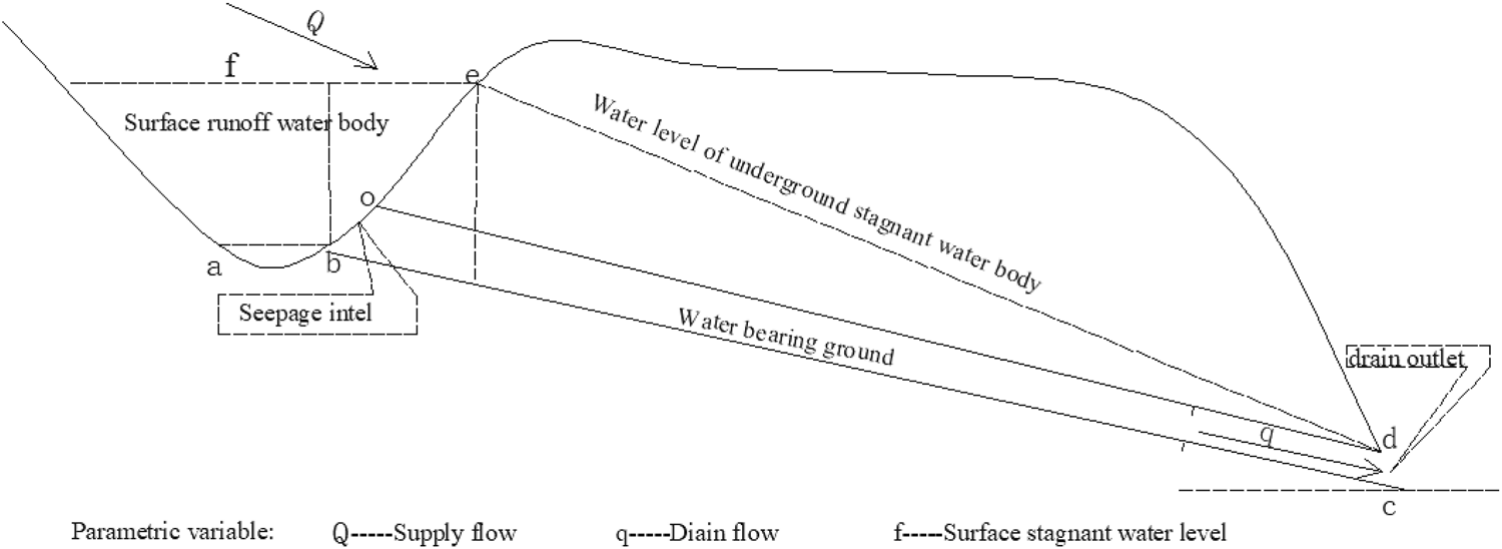
Illustrative diagram of the relationship among recharge flow, stagnation flow and seepage flow.

**Figure 3:**
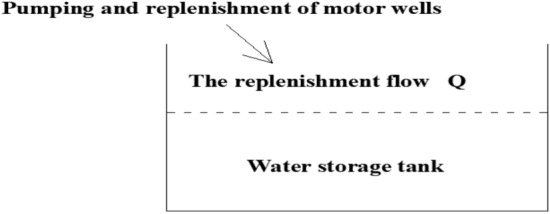
There is no drainage outlet and passage, and the water level in the reservoir rises continuously.

As shown in Fig. 2, there is a unified relationship among surface stagnant water, underground stagnant water, recharge water and seepage. According to Fig. 3, it can be seen that two physical quantities, seepage and stagnation, can exist simultaneously on any cross-section of water flow. Seepage flow and stagnation flow always constitute a pair of physical quantities with opposite behaviors and at the same time are unified with each other. This is the basic law of water flow.

In fact, the fluid movement phenomenon includes the following three equations of the law of unity of opposites.

### Equations of the law of unity of opposites of action

The reason why the fluid moves first depends on the driving action it receives. In this driving action, there is a law equation system of unity of opposites.

The driving action amount *A* generated by the driving action can not be used to drive the fluid to run, but only part is used to drive the fluid to run through the pore channel on a certain section, and this part of the driving amount is the virtual amount *A*_*F*_. The other part is the action quantity used to overcome resistance and compete with the action quantity of obstruction, it is transformed into the action quantity driving the rise of ponding water level under the control of resistance, which is the actual amount *A*_*T*_. The ratio of the virtual action amount *A*_*F*_ to the action amount *A* is equal to the virtual degree, that is, the virtual action rate *E.* The ratio of the actual amount *A*_*T*_ to the action amount *A* is equal to the reality, that is, the actual action rate *T*. *A*_*F*_ and *A*_*T*_ have opposite physical meanings and are considered to be opposite. Both *A*_*F*_ and *A*_*T*_ constitute *A* part of a respectively and have a unified relationship, that is, *A*_*F*_ and *A*_*T*_ are opposite and unified in *A*. *E* and *T* represent virtual action rate and real action rate respectively, and represent two different properties of fluid receiving action respectively. Their meanings are opposite, but they are both used to describe the properties of the same fluid section receiving the same action and have unified characteristics. *T* and *E* also have the law of unity of opposites: *T* and *E* are unity of opposites in 1. *A,* *A*_*T*_*,* *A*_*F*_*,* *E* and *T* are closely related and inseparable five important physical quantities. However, the unity of these five quantities cannot be expressed clearly by a single function, but must be expressed completely by a system of equations. The equations are expressed as follows:

9 $$\left\{ \begin{gathered} A_{F} + A_{T} = A \hfill \\ A_{F} = EA \hfill \\ A_{T} = TA \hfill \\ E + T = 1 \hfill \\ \end{gathered} \right.$$

This system of equations is called the system of unity of opposites. Since *F* = *A/t,* *F*_*F*_ = *A*_*F*_*/t,* *F*_*T*_ = *A*_*T*_*/t,* *F,* *F*_*F*_*,* *F*_*T*_ are represent force, virtual force and strength respectively, the equations of unity of opposites of action can also be expressed as Eq. (10).

10 $$\left\{ \begin{gathered} F_{F} + F_{T} = F \hfill \\ F_{F} = EF \hfill \\ F_{T} = TF \hfill \\ E + T = 1 \hfill \\ \end{gathered} \right.$$

This is the first basic law existing in fluid motion.

### The changing equation of unity of opposites

The essence of fluid movement is the change of fluid. The maximum change that the driving action can cause the fluid to change is called the change, which is recorded as *B.* The seepage variation formed by the fluid under the driving action is the virtual variation *B*_*F,*_ the stagnant variation produced by the fluid under the driving action is the real variation *B*_*T*_*.* The distribution relationship between *B*_*F*_ and *B*_*T*_ depends on the operation rate and delay rate. The operation rate is equal to the ratio of virtual change to change, which is equal to virtual *E.* The lag rate is equal to the ratio of the real change to the change, which is equal to the real degree *T*. The law of unity and unity of opposites among *B,* *B*_*T*_*,* *B*_*F*_*,* *E* and *T* also constitutes an equation group, which is called the change unity of opposites equation group:

11 $$\left\{ \begin{gathered} B_{F} + B_{T} = B \hfill \\ B_{F} = EB \hfill \\ B_{T} = TB \hfill \\ E + T = 1 \hfill \\ \end{gathered} \right.$$

This is the second basic law existing in fluid motion.

### Unified law equations between action and change

There is a law of mutual correspondence and unity between function and change, and there is always a corresponding amount of action corresponding to what kind of function there is, and there is always a corresponding amount of action corresponding to the amount of change. This unity is produced by the correspondence between the system of equations of the unity of opposites of action and the system of equations of the unity of opposites of variation, that is:

12 $$\left. \begin{gathered} B_{F} + B_{T} = B \hfill \\ B_{F} = EB \hfill \\ B_{T} = TB \hfill \\ E + T = 1 \hfill \\ \end{gathered} \right\} = \left\{ \begin{gathered} A_{F} + A_{T} = A \hfill \\ A_{F} = EA \hfill \\ A_{T} = TA \hfill \\ E + T = 1 \hfill \\ \end{gathered} \right.$$

This is the third basic equation system existing in the phenomenon of fluid motion.

### Basic research methods of fluid motion law

The three equations explain the basic laws and complete laws existing in fluid motion. There are four main steps in the study of fluid motion law. Here, taking the flow or groundwater seepage movement as an example, the basic methods and steps of fluid movement law research are as follows:

The first step is to study the role of measurement in controlling water flow.

The dual one-variable equation (Eq. 11) is introduced to observe and study the parameters in the dual one-variable equation in practical problems. Practice has proved that in the phenomenon of water flow or groundwater seepage, it is impossible to directly measure any of the five quantities of action *A*, virtual action *A*_*F*_*,* actual action *A*_*T*_*,* virtual action *E* and real action *T* in the equations. However, the action quantity or action function can be measured indirectly.

According to the actual observation and research, the driving amount leading to the flow movement is generated by the momentum of the recharge flow, gravity and the pressure of the stagnant water. The effects of these three sources are:

1. Action generated by recharge flow
  The momentum of the recharge flow can produce an action quantity to control the flow movement, and its value is closely related to the recharge flow *Q*. That is:
  13 $$A_{1} = \frac{{\rho Q^{2} t}}{S}$$
  In the equation, *S* represents the area of the section under the action of water flow; *A*_*1*_ represents the action amount generated by recharge flow *Q*; *P* represents the specific gravity of water flow; *t* represents the time of water flow movement. According to the equation, in practical problems, the action *A*_1_ value generated by the recharge flow can be determined by measuring the recharge flow *Q*, the affected section area *S*, the fluid specific gravity *P* and the flow time *t*. *A*_1_ value is actually the initial action amount of fluid acting on section *S.*
2. Driving action caused by gravity
  After the recharge flow *Q* enters the action system, it will accept the action of gravity, so as to obtain the action amount acting on the affected section *S*_*0*_, which is called gravity action amount, and its value is:
  14 $$A_{2} = \int_{0}^{t} {\rho Qgtdt}$$
  By measuring *Q* value and *P* value, *A*_2_ value can be obtained through calculation. *A*_2_ is the action increment caused by gravity.
3. Action caused by initial hysteresis flow
  The stagnant flow, such as the water volume in the upstream low-lying area and the water volume of the reservoir, can also produce an action quantity on the studied affected section *S*, such as the action quantity produced by the water level difference, which is closely related to the initial water level difference △*H* = *H*_1_ *−* *H*_2_, that is:
  15 $$A_{3} = \rho S_{S} \left( {H_{1} - H_{2} } \right)gt$$

In the formula, *S*_*S*_ represents the horizontal distribution area of stagnant water body; *H*_1_ and *H*_2_ represent the initial water level above and below section *S* respectively, and *H*_2_ = constant. *A*_3_ is the action amount generated by water pressure.

The total action of controlling water flow movement is equal to the sum of the above three actions, that is:

16 $$A = A_{1} + A_{2} + A_{3} = \frac{{\rho Q^{2} t}}{S} + \int_{0}^{t} {\rho Qgtdt} + \rho S_{S} \left( {H_{1} - H_{2} } \right)gt$$

This action quantity *A* is the action quantity determined on the basis of measured research, which is called measurable or known quantity. Since *A*_*F*_ = *EA* and *A*_*T*_ = *TA*, Then:

17 $$A_{F} = E\left[ {\frac{{\rho Q^{2} t}}{S} + \int_{0}^{t} {\rho Qgtdt} + \rho S_{S} \left( {H_{1} - H_{2} } \right)gt} \right]$$

18 $$A_{T} = T\left[ {\frac{{\rho Q^{2} t}}{S} + \int_{0}^{t} {\rho Qgtdt} + \rho S_{S} \left( {H_{1} - H_{2} } \right)gt} \right]$$

This leads to a new set of equations for the unity of opposites:

19 $$\left\{ \begin{gathered} A_{F} + A_{T} = A \hfill \\ A_{F} = E\left[ {\frac{{\rho Q^{2} t}}{S} + \int_{0}^{t} {\rho Qgtdt} + \rho S_{S} \left( {H_{1} - H_{2} } \right)gt} \right] \hfill \\ A_{T} = T\left[ {\frac{{\rho Q^{2} t}}{S} + \int_{0}^{t} {\rho Qgtdt} + \rho S_{S} \left( {H_{1} - H_{2} } \right)gt} \right] \hfill \\ E + T = 1 \hfill \\ \end{gathered} \right.$$

This system of equations is generated on the basis of actual measurement, which is called the system of unity of opposites of actual measurement.

The second step is to study and measure the change of water flow.

In flow or seepage, two always coexisting changes can be observed:

1. Flow or seepage phenomenon, i.e. virtual change phenomenon
  The measured flow through section *S* is *q.* The momentum of this flow is *I*_*t*_ = *pq*^*2*^*t/ES*. Where, *E* represents the void, which is equal to the porosity of section *S* under specific conditions; *S* is called the active section. The movement of water flow through section *S* belongs to the phenomenon of virtual change, and it can be regarded as a physical quantity used to measure the phenomenon of virtual change. Therefore, the virtual variation function is obtained by replacing *I*_*t*_ with the virtual variation symbol *B*_*F*_:
  20 $$B_{F} = \frac{{\rho q^{2} t}}{ES}$$
  *B*_*F*_ is the only measurable virtual change in seepage change phenomenon.
2. Change phenomenon of stagnant water
  Water flow or seepage is blocked to form stagnant water. The change of stagnant water is displayed by the change of stagnant water level. Considering the dimensional unity, the momentum unit is also used to measure the change of stagnant flow.

According to the measurement, the real-time water level of stagnant water is *H*_1*t*_ = *H*_1_ + *vt* (*v* is the rising and falling speed of stagnant water level, *t* is the change time, *vt* is eaual to the water level increment of stagnant water),. The water level downstream of section *S* is *H*_2_, and the horizontal distribution area of stagnant water is *S*_*S*_*.* According to the existing mechanical knowledge, it can be determined that the physical quantity equivalent to the change of stagnant water is:

21 $$B_{T} = \int_{0}^{t} {\rho S_{S} vgt} dt$$

According to the research, the variation of water flow is equal to the sum of virtual variation *B*_*F*_ and real variation *B*_*T*_, that is:

22 $$B = B_{F} + B_{T} = \frac{{\rho q^{2} t}}{ES} + \int_{0}^{t} {\rho S_{S} vgt} dt$$

On this basis, the measured equations of flow movement are obtained:

23 $$\left\{ \begin{gathered} B_{F} + B_{T} = B \hfill \\ B_{F} = \frac{{\rho q^{2} t}}{ES} \hfill \\ B_{T} = \int_{0}^{t} {\rho S_{S} vgt} dt \hfill \\ E + T = 1 \hfill \\ \end{gathered} \right.$$

The theoretical equations of water flow are as follows:

24 $$\left\{ \begin{gathered} B_{F} + B_{T} = B \hfill \\ B_{F} = EB \hfill \\ B_{T} = TB \hfill \\ E + T = 1 \hfill \\ \end{gathered} \right.$$

The third step is to study the unity between water flow movement and its control effect.

Due to the measured action, the unity of opposites equations and the change unity of opposites equations are as follows:

25 $$\left\{ \begin{gathered} A_{F} + A_{T} = A \hfill \\ A_{F} = E\left[ {\frac{{\rho Q^{2} t}}{S} + \int_{0}^{t} {\rho Qgt} dt + \rho S_{S} \left( {H_{1} - H_{2} } \right)gt} \right] \hfill \\ A_{T} = T\left[ {\frac{{\rho Q^{2} t}}{S} + \int_{0}^{t} {\rho Qgt} dt + \rho S_{S} \left( {H_{1} - H_{2} } \right)gt} \right] \hfill \\ E + T = 1 \hfill \\ \end{gathered} \right.\left\{ \begin{gathered} B_{F} + B_{T} = B \hfill \\ B_{F} = \frac{{\rho q^{2} t}}{ES} \hfill \\ B_{T} = \int_{0}^{t} {\rho S_{S} vgt} dt \hfill \\ E + T = 1 \hfill \\ \end{gathered} \right.$$

The corresponding equations in the two equations are equal, so the unified equations of action and change can be further established:

26 $$\left\{ \begin{gathered} A_{F} + A_{T} = A = B_{F} + B_{T} = B \hfill \\ A_{F} = E\left[ {\frac{{\rho Q^{2} t}}{S} + \int_{0}^{t} {\rho Qgt} dt + \rho S_{S} \left( {H_{1} - H_{2} } \right)gt} \right] = B_{F} = \frac{{\rho q^{2} t}}{ES} \hfill \\ A_{T} = T\left[ {\frac{{\rho Q^{2} t}}{S} + \int_{0}^{t} {\rho Qgt} dt + \rho S_{S} \left( {H_{1} - H_{2} } \right)gt} \right] = B_{T} = \int_{0}^{t} {\rho S_{S} vgt} dt \hfill \\ E + T = 1 \hfill \\ \end{gathered} \right.$$

This set of equations is used to describe the complete law of water flow movement, in which the unified law between various physical quantities is included in the set of equations.

The fourth step is to study and solve practical problems.

According to Eq. (25), two coexisting equations (Eqs. 27 and 28) can be further obtained through derivation:

27 $$E\left[ {\frac{{\rho Q^{2} t}}{S} + \int_{0}^{t} {\rho Qgt} dt + \rho S_{S} \left( {H_{1} - H_{2} } \right)gt} \right] = \frac{{\rho q^{2} t}}{ES}$$

28 $$T\left[ {\frac{{\rho Q^{2} t}}{S} + \int_{0}^{t} {\rho Qgt} dt + \rho S_{S} \left( {H_{1} - H_{2} } \right)gt} \right] = \int_{0}^{t} {\rho S_{S} vgt} dt$$

The equation of motion of the excretory flow:

29 $$q = E\sqrt {S\left[ {\frac{{Q^{2} }}{S} + Qgt + S_{S} \left( {H_{1} - H_{2} } \right)g} \right]}$$

Water level variation equation:

30 $$\Delta H = T\left[ {\frac{{Q^{2} }}{{SS_{S} g}} + \frac{Qt}{{S_{S} }} + \left( {H_{1} - H_{2} } \right)} \right] = vt$$

According to these two equations, on the basis of observation, through analysis and calculation, any unknown quantity in the equation can be obtained, so as to solve practical problems.

Through the above research, we know that the driving effect of controlling fluid movement actually comes from gravity, and it is gravity that dominates the operation or seepage of the fluid. The running channel and the obstacle material constitute the restrictive factors that restrict the operation of the fluid or seepage. Gravity gives the fluid the power to run, but whether it runs depends on whether there is a running channel: if there is a channel, it will run; if there is no channel, it will stop running; if there is a channel but there is a resistance limit, there will be both running flow and stagnant water level increment. This is the basic mechanism of fluid motion.The seepage channel is the spatial channel through which the water flows. It can run on the channel, but the resistance needs to be overcome. There may be obstacles on the channel, but it can run through. The obstacle only prolongs the running time and reduces the running speed.The supplement is as follows:

As shown in Fig. 3, for the convenience of water, the water from the motor well is pumped to the reservoir. Because there is no drainage outlet and passage in the reservoir, the water level in the reservoir continues to rise, there is no drainage flow, only the water level rises and changes.

**Figure 4:**
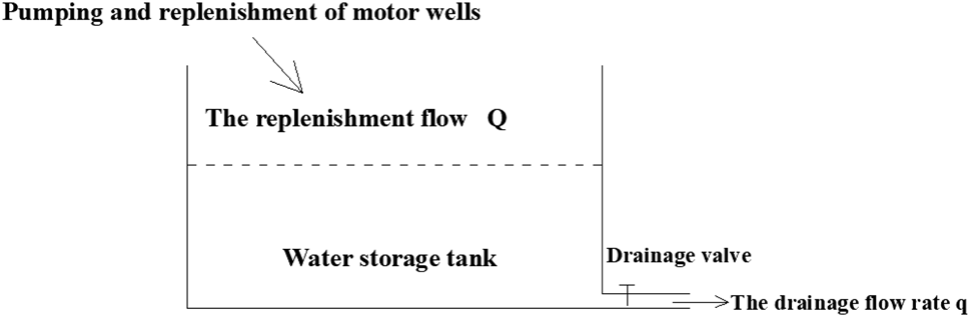
There is a drainage outlet and passage, and the drainage valve controls the drainage resistance.

As shown in Fig. 4, a drain pipe is installed in the reservoir, and a discharge control valve is installed on the drain pipe, which can control the discharge. The working principle of water valve to control displacement is to control displacement by increasing or decreasing resistance. When the resistance increases to the limit, the outlet is completely sealed, the discharge is equal to zero, only the water level rises in the pool, there is no discharge; reduce the drainage resistance, that is, increase the outlet area of the drainage valve, there will be drainage flow. When the displacement is less than the recharge flow, the water level in the reservoir is also rising, but the rising speed becomes smaller. When the displacement is greater than the recharge flow, the water level in the reservoir will continue to drop.

## Experimental study on new equation of seepage theory

Seepage operation Eq. (29) and stagnant water level rise and fall calculation Eq. (30). It is an equation used to clarify the unified relationship between flow movement and hysteresis. As long as the two equations are proved to be correct, it can be proved that the unified relationship between movement and hysteresis is correct.

**Figure 5:**
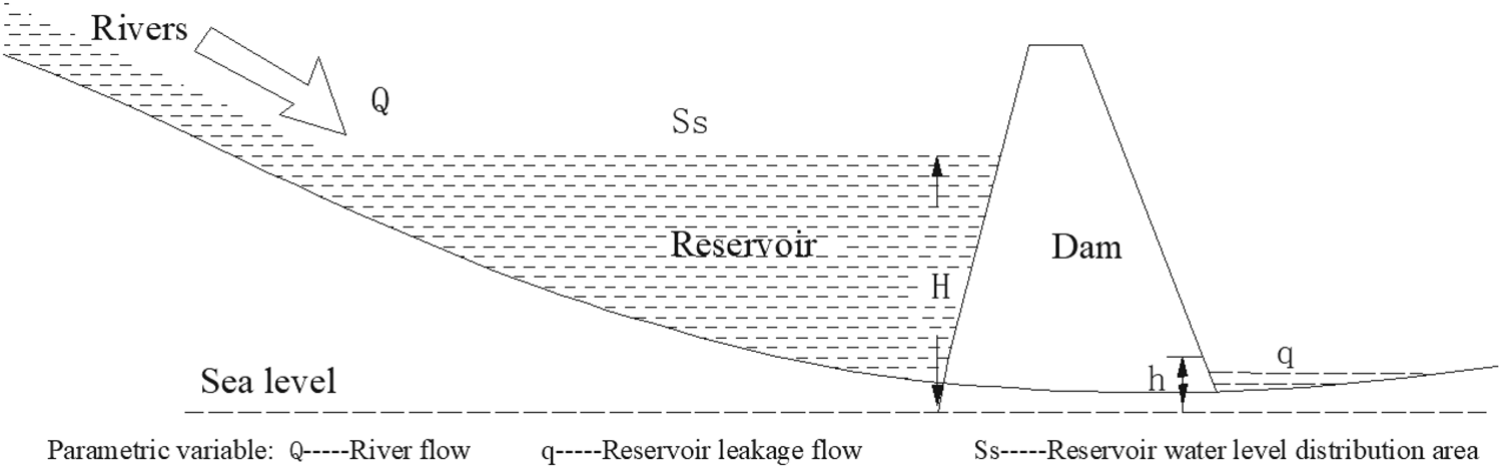
Schematic diagram of unified experiment of flow and hysteresis.

As shown in Fig. 5, there is a river on the surface. Building a large water retaining dam in the river and building an experimental structure integrating seepage and stagnation. Among them, the river flow to the reservoir is *Q* = 30 m^3^/h; the leakage flow of the reservoir is *q*; the leakage section area of the dam is *S*; the porosity of *S* is *E* = 0.012; the initial water level after the completion of the reservoir is *H*_1_ = 2 m; the rising speed of reservoir water level is *v.* The water seepage from the dam to the downstream is blocked and the formation water level *H*_2_ = 1 m, which is a constant. The distribution area of reservoir water level is *S*_*S*_ = *f* *(H)*; the running distance of dam seepage through section *S* is *x*.

Calculate the seepage flow and the water level increment in a period of time, and demonstrate the correctness of Eqs. (29) and (30).

Solution: from Eq. (29):

31 $$q = 0.012\sqrt {S\left[ {\frac{{30^{2} }}{S} + 30 \times 9.8t + 9.8f\left( H \right)\left( {2 - 1} \right)} \right]} = 0.012\sqrt {900 + 294t + 9.8f\left( H \right)}$$

From *E* + *T* = 1 come to the conclusion *T* = 1–0.012 = 0.918.

From Eq. (30):

32 $$\Delta H = 0.918\left[ {\frac{{30^{2} }}{9.8Sf\left( H \right)} + \frac{30t}{{f\left( H \right)}} + 1} \right] = vt$$

Demonstration: first of all, the calculated data *q* and *∆H* = *vt* are obtained through calculation, and the observed data *q* and ∆*H* = *vt* are obtained through observation. Then, the observed data are compared with the calculated data. The results of several comparisons show that the calculated datas are completely equal to the observed datas. It is proved that Eqs. (29) and (30) are correct.

## Comparative study with previous seepage research methods

The previous research on the relationship between seepage and action is mainly Darcy's law. Although Darcy's law is based on experiment, its conclusion is mainly limited to basic understanding. That is, on the basis of believing that the seepage volume is directly proportional to the water level difference, directly proportional to the seepage cross section and inversely proportional to the seepage distance. The Darcy law is established by using the parameter introduction method.

33 $$q = k\frac{H - h}{{\Delta L}}S$$

In the formula, *Q* is the seepage flow; *H–h* = *△H* is the water level difference; S is the seepage section; *△L* is the seepage distance.However, by comparing and calculating, Eqs. (29) and (33) are different.

It can be seen that there are still some problems in the previous quantitative scientific research.

## How to consider turbulence

Both fluid mechanics and percolation mechanics discuss advection, turbulent flow. The core issues involved in this paper are the exact and overall laws of fluid motion, while advection and turbulence issues are secondary issues, which cannot be discussed too much due to space limitations. But it must be pointed out that: no matter advection or turbulent flow, its operation law obeys the law stated by the Eq. (29).

Whether the fluid moves in advection or turbulent motion is controlled by only two factors: the size of the driving force and the nature of the channel. The driving force is related to the recharge flow, seepage section, stagnant water level area, water level difference, and gravitational acceleration, that is

34 $$F = \frac{{F_{F} }}{E} = \frac{{\rho \left[ {Q^{2} + SQgt + SS_{S} \left( {H_{1} - H_{2} } \right)g} \right]}}{S}$$

The channel property parameters are related to the channel section size, channel smoothness, porosity and other factors. Generally speaking, when the driving force is large and the running channel is rough, the fluid will move in the form of turbulent flow. However, no matter how it operates, the basic laws of fluid operation remain unchanged.

## Conclusion

From the above analysis and research, the following conclusions can be drawn:

1. The movement of fluid or seepage obeys the law of unity of opposites and action, the law of unity of opposites and change, and the law of unity of action and change. There is a law of unity of opposites between seepage and stagnation (stagnant water).
2. A single equation cannot accurately and completely describe the motion law of fluid or seepage. Only the unified equations of opposites can accurately and completely describe the law of fluid or seepage motion.
3. There is a direct functional relationship between seepage and recharge flow, seepage cross-sectional area, gravitational acceleration, water level difference, and stagnant water distribution area. But there is no direct functional relationship between seepage flow and flow or seepage distance. The increase or decrease of the retained water level is directly related to the recharge flow, the seepage cross-sectional area, the water level difference, the distribution area of the water level difference, the acceleration of gravity, and the seepage time.
4. The parameter introduction method is not suitable for percolation research. The research on the law of seepage movement must use the scientific method determined by the natural law itself. The method of natural science is determined by the unified equations of action opposites, the unified equations of change, and the unified equations of action and change.
5. Fluid running mechanism: gravity gives the fluid the power to run, but whether it runs depends on whether there is a running channel: if there is a channel, it will run; if there is no channel, it will stop running; if there is a channel but there is a resistance limit, there will be both running flow and stagnation flow level increment.

## Data Availability

The datasets used and/or analysed during the current study available from the corresponding author on reasonable request.
